# Towards Identify Selective Antibacterial Peptides Based on Abstracts Meaning

**DOI:** 10.1155/2016/1505261

**Published:** 2016-06-05

**Authors:** Liliana I. Barbosa-Santillán, Juan J. Sánchez-Escobar, M. Angeles Calixto-Romo, Luis F. Barbosa-Santillán

**Affiliations:** ^1^University of Guadalajara, Guadalajara, JAL, Mexico; ^2^Technical and Industrial Teaching Center, Guadalajara, JAL, Mexico; ^3^The College of the South Border (ECOSUR), Tapachula, CHIS, Mexico

## Abstract

We present an Identify Selective Antibacterial Peptides (ISAP) approach based on abstracts meaning. Laboratories and researchers have significantly increased the report of their discoveries related to antibacterial peptides in primary publications. It is important to find antibacterial peptides that have been reported in primary publications because they can produce antibiotics of different generations that attack and destroy the bacteria. Unfortunately, researchers used heterogeneous forms of natural language to describe their discoveries (sometimes without the sequence of the peptides). Thus, we propose that learning the words meaning instead of the antibacterial peptides sequence is possible to identify and predict antibacterial peptides reported in the PubMed engine. The ISAP approach consists of two stages: training and discovering. ISAP founds that the 35% of the abstracts sample had antibacterial peptides and we tested in the updated Antimicrobial Peptide Database 2 (APD2). ISAP predicted that 45% of the abstracts had antibacterial peptides. That is, ISAP found that 810 antibacterial peptides were not classified like that, so they are not reported in APD2. As a result, this new search tool would complement the APD2 with a set of peptides that are candidates to be antibacterial. Finally, 20% of the abstracts were not semantic related to APD2.

## 1. Introduction

The knowledge acquired in the discovery of the antibacterial peptides has been essential to the longevity of the human being. However, many of these findings have suffered changes. One of them was the flu [1] immunity case presented to the antibiotics.

A global problem is the attack to the bacteria with new antibiotics since more and more deaths exist for infectious illnesses that earlier were treated by the existing antibiotics.

As a consequence, it is necessary to identify new antibacterial peptides that have been approved and tested by experts but are not labeled as such.

Fortunately, the scientists, laboratories, and universities are describing new peptides in research articles continuously. Unfortunately, these grow exponentially [2] and the peptides description is heterogeneous.

One of the great advances of the 21st century has been the transfer of scientific discoveries through open access sites such as PubMed [3]. However, to identify the articles that have antibacterial peptides in more than 10,000 records is not a trivial task.

One option is to purge the databases manually based on the peptides amino acid sequences as APD2 [4] and Novković et al. [5]. APD2 has 1913 and Novković et al. has 2571 antibacterial peptides, respectively. These include the antibacterial peptides sequence and some of their properties such as activity, source, author, and method.

One of the challenges identified in peptide sequence-based search is the zero possibility of recovering neuropeptides. In this sense, antibacterial sequences already bring a bias that does not include neuropeptides; other peptides that produce living beings could also be antibacterial.

The difficulties of our research are (a) to relate automatically the peptides abstracts of the articles in a semantic way and (b) to identify peptides that are not classified as antibacterial by the experts because they have only tagged one part. Our main contribution is the identification and prediction of current and new antibacterial peptides abstracts, so pharmaceutical companies and laboratories will develop antibiotics of the next generation. Our research questions are as follows:

(Q1) Is it possible to identify antibacterial peptides automatically into abstracts of research articles in PubMed that have been annotated in APD2?
(Q2) Is it possible to predict antibacterial peptides automatically into the articles of databases such as PubMed that have not been annotated in APD2?
(Q3) How to identify and predict Selective Antibacterial Peptides based on its semantics?

Our research hypotheses are as follows:

(H1) It is possible to discover peptides which have not been classified as antibacterial in abstracts of research articles based on semantics.
(H2) It is possible to have a set of peptides that are candidates to be antibacterial but which have not been tagged as such.

The research of Wang et al. reported the APD [4] creation. It allows identifying peptides by key words; as a result peptides with biological activity such as antibacterial, anticancer, and antifungal are shown.

 Shtatland et al. [6] included in their database some of the abstracts that report a peptide sequence in UniProt, PubMed, and APD2. In both cases, the peptide sequences search criterion is used. Compared with previous work, the major contribution of this paper is the following:

- Focusing on semantics to find and predict antibacterial peptides that are in the literature using a blind approach, that is, without knowing the antibacterial peptide sequence.

We propose Identify Selective Antibacterial Peptides (ISAP) approach. The ISAP relevancy is the capacity to identify and predict antibacterial peptides regardless of the sequence. That is based on the text content related to antibacterial peptides (APD2); our ISAP can learn excluding sequences of peptides of these texts and go for using and expanding their knowledge base.

Our approach consists of a semantic analysis where ISAP learns of the textual content, the frequency of words, and the antibacterial signatures or patterns. ISAP focuses on abstracts of the articles because these are an isolated and autonomous unit. Abstracts describe a problem, the main objectives, the investigation scope, the methods used, a summary, and the results and set out the conclusions. Scientists could not carry on up to date in their research fields without abstracts. Unfortunately, a variety exists in nomenclatures and words used by the authors when they describe their findings.

Due to the fact that they are from different parts of the world, peptides are formed with their amino acid codes located in standard tables. The authors can express them through a name, a code of one or multiple letters, or the d-amino acid code, among others.

The main objectives of ISAP approach are (a) to find antibacterial peptides automatically in PubMed that were annotated in APD2 and (b) to predict antibacterial peptides automatically that were not annotated in APD2. ISAP is important and original because many peptides have traditionally been studied outside of the context of bacteria. For example, peptides occurred in the nervous system could also be identified as antibacterial. A sequence-based search would not return any of the neuropeptides because the antibacterial sequences known already bring a bias.

Antibacterial peptides have been identified by calculating physicochemical properties in more than 12000 abstracts that had not been annotated as such in PubMed; these were considered only neuropeptides [7]. Recently, more neuropeptides have also been seen that may be antibacterial. However, this is not limited to neuropeptides; other peptides that produce living beings could also be antibacterial.

To use a semantic approach to identify the meaning of the words (excluding the peptides sequences) in a PubMed articles sample allows relating the antibacterial peptides work and could complement the physicochemical properties calculation. For example, many antibacterial peptides are cationic and amphipathic; these properties are also present in penetrators peptides cells and some neuropeptides, among others.

ISAP is trained to recognize antibacterial peptides abstracts excluding the peptide sequences that we called a ISAP blind approach. We have validated ISAP by matching antibacterial peptides abstracts found by the ISAP approach in the APD2 database. In other words, we have given an abstract of an article with content linked to peptide. ISAP is able to know if it is antibacterial or not. If ISAP concludes that it is true, then it classifies it as antibacterial. ISAP correlates its result with the APD2 database. If the correlation is equal to one, then it is true; otherwise it is false. The problem of finding and predicting antibacterial peptides in engines such as PubMed is a BigData issue because it contains the four “V”s: velocity, variety, volume, and veracity.

1. *Velocity*. The speed of the authors publishing their results is exponential [2].
2. *Variety*. The number of countries, researches, authors, languages, and results is heterogeneous.
3. *Volume*. Each 10 seconds [2] has a new article, so we are talking about 80 terabytes of data until 2014 in just one database, but there are more than 20 recognized as PubMed, Protein, BioSample, and so forth.
4. *Veracity*. The veracity theoretically is true because ISAP is analyzing scientific results.

In addition, humans are susceptible to microbial infections that attack different parts of the body as urinary tract, ears, eyes, stomach, skin, lungs, liver, mouth, respiratory system, and reproductive systems. Such infections can destroy human life, so they must be fought mainly with antibiotics, which destroy the bacterial flora from different parts of the body so that antibiotics disrupt the mutuality or symbiotic relationship between bacteria and human body; intestinal bacterial flora is the most affected showing damages in the absorption of nutrients.

For this reason, many researchers around the world are interested in finding new antibiotics that do not affect human health. Antimicrobial peptides are a natural alternative with huge potential to replace conventional antibiotics that are marketed today.

Search for antimicrobial peptides could be limited to the in silico analysis in databases of reported protein sequences, such as those found in UniProt KB/Swiss-Prot protein knowledge bases where 549.832 protein sequences or microbial peptides sequences are reported in APD (http://aps.unmc.edu/AP/main.php) which have 2649 antimicrobial peptides, classified as antibacterial, antifungal, antiparasitic, anticancer, antiprotist, insecticidal, and so forth.

As a result of this massive data generation, computer science has become indispensable for biological research trying to uncover discriminatory patterns, such as sequences and three-dimensional structure but are not taking into account the semantics used in article abstracts of antimicrobial peptides found in several parts of the body, and they have not been classified as antimicrobial yet.

The remainder of the paper is structured as follows.  Section 2 briefly presents the background of our work.  Section 3 describes the Identify Selective Antibacterial Peptides (ISAP) approach.  Section 4 presents details of our data sets, evaluation metrics, and the result. Finally, Sections 5 and 6 present our discussion and conclusion.

## 2. Identify Selective Antibacterial Peptides (ISAP) Approach

The ISAP approach consists of four components which are divided into two stages: training and discovering. For the first stage there are three components: (1) GetPatterns, (2) GetPatterns-abstract collection, and (3) the Latent Semantic Indexing (LSI) process of the patterns-abstract collection. For discovering the antibacterial peptides there is one component: (4) the process of finding and predicting peptides-antibacterial abstracts. We will describe each one of these.

### 2.1. Training

Supervised learning is used in order to build ISAP knowledge base. It has information linked to the antibacterial peptides abstracts of articles. These were endorsed by experts and published in APD2 database.

The knowledge base process is essential for training. It consists of three stages: (a) to get antibacterial peptides of APD2 database, (b) to get GetPatterns-abstract collection from PubMed with a one-to-one relation with APD2, and (c) to index the patterns-abstracts collection.

#### 2.1.1. GetPatterns

Our first assumption is as follows: A1: there are peptides with antibacterial activity annotated by experts which are available on the web. GetPatterns is a crawler of antibacterial-peptides patterns.

It uses a parser in order to identify the sequences of antibacterial peptides. Its main objective is to get a list of peptides and its abstract related to APD2.

#### 2.1.2. GetPatterns-Abstract Collection

Public electronic databases such as PubMed Central, through the Open Archive Initiative-Protocol Metadata Harvesting (OAI-PMH) with the PMC-OAI service, provide access to metadata of all items. OAI-PMH is a standard protocol for the compilation of metadata. This was designed to share records in an open and free way which is promoted by the Open Archive Initiative community. OAI-PMH is based on the exchange of messages in XML on HTTP services.

GetPatterns-abstract collection makes connections to the OAI-PMH service to download the metadata and its content. The resulting instances are in simplified Dublin Core format. For each obtained instance a transformation of the Dublin Core format to the text file is performed. The text file has the following metadata: title, authors, abstract, year, journal, and id assigned by the PMC-OAI service. The antibacterial peptide patterns filter is used to obtain the abstracts of articles with a one-to-one relation with APD2.

From the past ten years sorted in descending order, in this search we found that the PubMed Central site has more than 342,821 peptides articles. However, ISAP only gets related abstracts to APD2 reducing the sample to 1800 abstracts linked to antibacterial peptides. The rest was discarded because ISAP approach test was conducted with a random selection sample carried out with a 5% of the articles linked with peptides content in PubMed between the years 1999 and 2011, respectively.

#### 2.1.3. Latent Semantic Indexing (LSI) Process

The LSI process allows us to index the patterns-abstract collection in order to build a knowledge base in the following way.

The abstracts of scientific articles are processed one by one in a local directory. Then, two lists are created, one with the name of each existing article and another with the main words that belong to the article. Both have their respective PubMed unique identifiers (PMIDs).

The list of words is pruned to eliminate those that have no value. They are compared with a stop-words list and a process of stemming is applied in order to get the morphological root of each word.

The terms-documents matrix is generated with the words list and files. The columns represent the pattern-abstract corpus and rows represent the words.

Each cell in the matrix is assigned a score. First, this is obtained by the frequency with which every word appears in every document. Then, a cosine distance function of each cell is performed in order to normalize the data and thus a Weighted Term-Document Matrix (WTDM) is obtained.

The matrix operation of singular value decomposition (SVD) is applied to the Weighted Term-Document Matrix to get the submatrices *U*, *S* (diagonal) and *V*. Setting the appropriate value of *k* allows us to reduce the dimension of the *U*, *S*, and *V* matrices. The *U*_*k*_, *S*_*k*_, and *V*_*k*_ matrices are known as reduced SVD.

The *S*_*k*_^−1^ matrix and the transposed *V*_*k*_ matrix (*V*_*k*_^*T*^) are calculated. Finally, all these matrices, WTDM, *U*_*k*_, *S*_*k*_^−1^, and *V*_*k*_^*T*^, are stored on disk in a binary file to be used in the processes of finding and predicting the antibacterial peptides abstracts in the knowledge base.

### 2.2. Discovering

Given the abstract of an article (search expression) it is known whether or not antibacterial through three rules:

(R1) If the result of ISAP between the search expression and patterns-abstract collection is greater than 85%, it is antibacterial.
(R2) If the result of ISAP between the search expression and patterns-abstract collection is less than 80%, it is not antibacterial.
(R3) If the result of ISAP between the search expression and the patterns-abstract collection is greater than 80% and less than 85% ISAP, it is predicted to be antibacterial.

**Figure 1:**
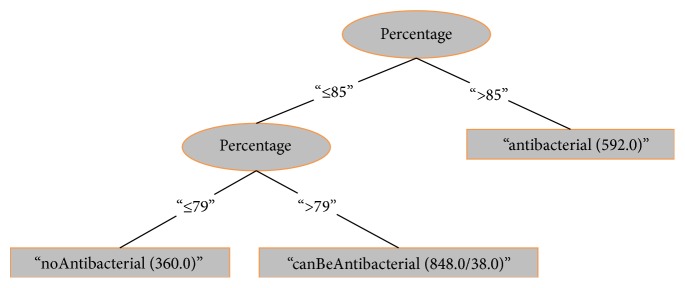
(R1) percentage > 85: antibacterial (592.0), (R2) percentage ≤ 85 and percentage ≤ 79: noAntibacterial (360.0), and (R3) percentage ≤ 85 and percentage > 79: canBeAntibacterial (848.0/38.0).

These values were determined with an empirical validation. The supervised method used is J48 decision trees as shown in Figure 1 where the number of leaves is 3 and the size of the tree is 5.

#### 2.2.1. The Process of Finding and Predicting Peptides-Antibacterial Abstracts

The process of finding and predicting the articles based on the degree of similarity to the search expression with ISAP knowledge base has six steps, as described below.

The information previously processed in LSI (i.e., the semantic space represented by the reduced SVD matrices) is stored in a binary file and loaded into memory for use in the search process.

All the words that make up a search expression are processed in a similar way to the ISAP knowledge base. First, the words are filtered using the stop-words list. Then, the stemming process is applied in order to convert each word to its root.

A query-vector (*q*_*T*_) is generated with the result of the process to the query words. *q*_*T*_ is normalized by applying the cosine formula and then the multiplication operations *q*_*T*_ *∗U*_*k*_ *∗S*_*k*_^−1^ are performed in order to get the final query-vector.

Meanwhile, the query-document is created. It is used to compare its similarity on latent semantic space with the query-vector. For every abstract a *a*_*T*_ (the transposed vector of abstracts) vector is created with the WTDM matrix information. The following operations are applied: *a*_*T*_ *∗U*_*k*_ *∗S*_*k*_^−1^ in order to get the vector-document.

Finally, the similarities between the candidate abstract and ISAP knowledge base is calculated. This is possible by applying the formula of the cosine distance between the query-document and query-vector, as shown in (1). The result is stored in a vector of similarities ranking, where each cell represents the similarity value of each abstract ISAP knowledge base with respect to the target abstract:

(1) $$\begin{array}{c} \cos\left(\;q_{T},a_{T}\;\right)\dot{≔}\left\{\;\frac{\left(\sum_{k=1}^{m}w_{1,k}\cdot w_{2,k}\right)}{\left(\sqrt{\sum_{k=1}^{m}w_{1,k}^{2}}\cdot \sqrt{\sum_{k=1}^{m}w_{1,k}^{2}}\right)}\;\right\}. \end{array}$$

The results of the similarity vector are with three possible answers. (a) The article has semantic content related to an antibacterial peptide, so it is an antibacterial peptide. (b) The article has no semantic content related to antibacterial peptide; therefore ISAP concludes that it is not antibacterial. (c) The article has semantic content related to antibacterial peptides but is not included in APD2, so ISAP predicts that it is antibacterial.

## 3. Illustration

The Identify Selective Antibacterial Peptides (ISAP) approach aims to identify and predict peptides with antibacterial activity that are in the literature based on its semantics. For that goal, the method obtained a corpus composed of abstracts from scientific articles endorsed by magazines and published in PubMed with a one-to-one relation in the APD2.

ISAP model is able to identify or predict whether it is or not antibacterial. In the first case, if the similarity between the abstract of article base and the knowledge base is greater than 85%, then ISAP identifies that the article has information about an antibacterial peptide. In the second case, ISAP predicts that the article has information about a peptide that can be antibacterial because there exists a semantic relation up to 80% and less than 85% with the model.

Besides, if the semantic similarity is lower than 80%, then ISAP concludes that the abstract does not have any information about an antibacterial peptide.

ISAP trains the model with the peptides subset which have been reported as antibacterials and has linked an article in the Antimicrobial Peptides Database 2 (APD2) until March 1, 2014. As a result, ISAP creates its knowledge base. It is learning in a semantic way with each of the abstracts of articles in APD2. Furthermore, ISAP discovers if one abstract has information that allows ISAP to decide whether it is antibacterial or not based on its semantic similarity related to the models knowledge base and the abstract of the article base.

On top of that, results are a knowledge base with papers' abstracts that belong to the APD2 with antibacterial activity.

To illustrate the use of ISAP approach we are going to run the methodology.

First, for the article A into ISAP, the goal is to discover through an identification or prediction if it is semantically related to a antibacterial peptide.

ISAP compares the distance between an isolated unit of knowledge as entry and the set of patterns of knowledge base. The more close the distance means that it is antibacterial.

The results are abstracts related to antibacterial peptides that are included in PubMed database but are not in APD2 database.

In summary, ISAP aim is to automate the process of cleaning databases in order to increase the global entries.

On the other hand, ISAP has two stages: (1) training and (2) discovering.

The first stage of ISAP is very important because it is where the abstracts patterns linked to antibacterial peptides has been learned. ISAP model has been training with 1991 and 2011 samples.

Less activity of antibacterial peptides was reported in 1991 in the literature in a sample of 5% of the entire corpora in spite of the fact that antibacterial peptides increased by 20% in 2011.

Next, ISAP runs each of the patterns of the knowledge base by performing semantic analysis in order to have metrics of the similarities between them and the abstract target.

Then, a vector space is generated where each of the abstracts is analyzed and measured in relation to cosine angle and the objective abstract; the more the small is the angle the semantically close is it and if it is negative, the opposite exists.

In the first stage of training, all abstracts related to patterns are the knowledge in the training process. ISAP learns from each of them in order to have a domain model. Then GetPatterns and GetPatterns-abstract collection are described as follows.

GetPatterns searched for the antibacterial peptides of the website (“http://aps.unmc.edu/AP/”) which were annotated manually. GetPatterns found the antibacterial peptides and execute the parser process in order to get the IDs and the peptide sequences that were stored in the patterns file. The result was 1762 antibacterial-peptides patterns, as shown in Table 1 and in Figure 1.

Each one of these corresponds to a peptide that has antibacterial activity in peptides-antibacterial sequences format.

Getting patterns-abstract articles collection resulted in 1800 scientific abstracts of articles obtained with the harvester OAI-PMH service. These were sorted according to the PMID abstract and these have a one-to-one relationship with the patterns.

One example of a peptides-article is shown in Box 1.

### 3.1. Latent Semantic Indexing (LSI) Process

LSI used a classical factorisation method from singular value decomposition to create a subspace in which research abstracts of articles were represented as vectors. First, a stop-word list was built with 747 terms that do not provide valuable information as they appear in the following equation:

(2) $$\begin{array}{c} IV\dot{≔}\left\{\begin{array}{c} and,not,the,\\ rp,with,those,\\ at,against,if,\\ among,anybody,around,aside\\ ask,associated,available,became,\\ because,anyways,apart,appear.\\ appreciate,around,available,\ldots \end{array}\right\}. \end{array}$$

Second, the terms-abstracts matrix was generated with 8892 terms that contain data related to the abstracts of peptides-antibacterial patterns, as shown in Table 2. As a result, our approach resulted in a lexicon of 8892 terms taken from the entire collection. Next, each cell had the value assigned by the frequency and normalization of all the terms through the cosine formula. The result was the Weighted Term-Document Matrix (WTDM).

Because the number in the relationship terms-abstract was huge, it was necessary to look for a dimension smaller than 100%. The optimal value of *k* was found to be 55% of the antibacterial patterns-abstracts collection.

Finally, all these matrices—WTDM, *U*_*k*_, *S*_*k*_^−1^ and *V*_*k*_^*T*^—are stored on disk in a binary file because they will be used in the process of finding and predicting peptides-antibacterial in the patterns-abstract collection. A partial view of the *S* matrix is shown in Box 1.

In addition, the Latent Semantic Indexing time was 20 minutes in an ASUS computer with a CPU Intel Core i7 a 2.3 GHz and 12 GB RAM.

*A Partial View of the S Matrix*

(3) $$\begin{array}{c} \begin{array}{c} \begin{array}{c} \left|0.276607067709848,0,0,0,0,0,0,0,0,0,0,0,0,0,0,0,0,0,0\right|\\ \left|0,0.366650066765251,0,0,0,0,0,0,0,0,0,0,0,0,0,0,0,0,0\right|\\ \left|0,0,0.382141723289962,0,0,0,0,0,0,0,0,0,0,0,0,0,0,0,0\right|\\ \left|0,0,0,0.411045008824079,0,0,0,0,0,0,0,0,0,0,0,0,0,0,0\right|\\ \left|0,0,0,0,0.418252506750572,0,0,0,0,0,0,0,0,0,0,0,0,0,0\right|\\ \left|0,0,0,0,0,0.423676370328037,0,0,0,0,0,0,0,0,0,0,0,0,0\right|\\ \left|0,0,0,0,0,0,0.459581947439162,0,0,0,0,0,0,0,0,0,0,0,0\right|\\ \left|0,0,0,0,0,0,0,0.468858032167802,0,0,0,0,0,0,0,0,0,0,0\right|\\ \left|0,0,0,0,0,0,0,0,0.478252119959795,0,0,0,0,0,0,0,0,0,0\right|\\ \left|0,0,0,0,0,0,0,0,0,0.503851970098151,0,0,0,0,0,0,0,0,0\right|\\ \left|0,0,0,0,0,0,0,0,0,0,0.511664251432095,0,0,0,0,0,0,0,0\right|\\ \left|0,0,0,0,0,0,0,0,0,0,0,0.533401538776504,0,0,0,0,0,0,0\right|\\ \left|0,0,0,0,0,0,0,0,0,0,0,0,0.541541268650656,0,0,0,0,0,0\right|\\ \left|0,0,0,0,0,0,0,0,0,0,0,0,0,0.548279729534182,0,0,0,0,0\right|\\ \left|0,0,0,0,0,0,0,0,0,0,0,0,0,0,0.567399790444048,0,0,0,0\right|\\ \left|0,0,0,0,0,0,0,0,0,0,0,0,0,0,0,0.571925860192531,0,0,0\right|\\ \left|0,0,0,0,0,0,0,0,0,0,0,0,0,0,0,0,0.573460335854454,0,0\right|\\ \left|0,0,0,0,0,0,0,0,0,0,0,0,0,0,0,0,0,0.575445844786161,0\right|\\ \left|0,0,0,0,0,0,0,0,0,0,0,0,0,0,0,0,0,0,0.578438845257281\right| \end{array} \end{array} \end{array}$$

### 3.2. Discovering

In this second phase, ISAP discovers if it is antibacterial or not, concluding three possible answers: it is, it is not, or it is likely to be.

#### 3.2.1. The Process of Finding and Predicting Peptides-Antibacterial Abstracts

This section explains the process of finding and predicting peptides-antibacterial in the patterns-abstract collection. First, the semantic structure of an abstract is represented as a vector and the degree of similarity between abstracts is calculated by the cosine distance of the angle between research-abstracts vectors.

Second, the relevance to the abstract is determined by ranking the similarity score, which is defined by the angle between the query and the patterns-abstract collection through the cosine formula.

### 3.3. The Model Validation

The model validation part was test performed with mode: 10-fold cross-validation. Therefore, total average accuracy of classification is 97% and misclassification or error rate is 2.11% as shown in Table 3.

Detailed accuracy by class for experiments with 1800 abstracts linked to antibacterial peptides is as shown in Table 4.

The classification done with all classes is as shown in confusion matrix in Table 5.

## 4. Results

The ISAP approach was tested with 253 peptides patterns. Five of them were selected randomly to be explained in this section as shown in Table 6.

Each of the patterns represents an experiment whose goal was to look for the presence of their peptide sequence in the research articles collection. One of the results was a peptides-article collection with 399 abstracts that were identified as having antibacterial-peptides patterns.

Finally, the knowledge base has PubMed unique identifiers (PMIDs) associated with the titles of the 399 peptides articles that contain any of the 238 antibacterial-peptides patterns in the abstract.

We will describe each one of the five patterns randomly selected.

**Figure 2:**
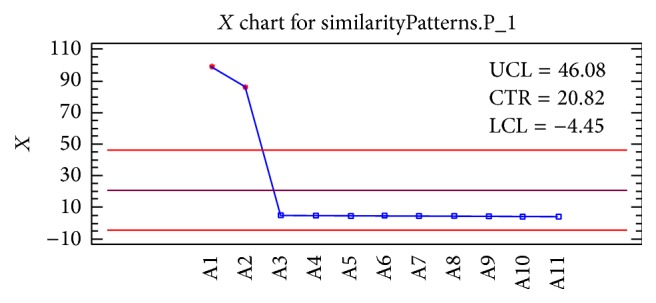
The results of the first pattern “AACSDRAHGHICESFKSFCKDSGRNGVKLRANCKKTCGLC.”

*Pattern 1*.  Figure 2 shows the results of the first pattern “AACSDRAHGHICESFKSFCKDSGRNGVKLRANCKKTCGLC.” We can observe that if the search expression has fewer terms, then there is less similarity with respect to the whole abstract. However, because the search process was semantic, the ISAP approach produced a collection of antibacterial peptides that match the first pattern.

For example, the article “PMID: 20362606” corresponds to “A defensin antimicrobial peptide from the venoms of* Nasonia vitripennis.*” It has the pattern content explicitly in the abstract.

The sequence of peptides and associated words was found im the following articles that are in PubMed but not Tossi DB: “PMID: 16890198, 15527787, and so forth.”

For each one of them, ISAP searched sequences of the antibacterial peptides in order to compare them with the predictions that are using the QSAR approach.

*Pattern 2*. The ISAP approach processed the second pattern “GLFDVIKKVASVIGGL.” The search was based on their content from the semantic point of view. As a result, Table 7 shows the research articles obtained in a list recorded in descending order.

*Pattern 3*. The article content associated with the PMIDs “22450466” related explicitly to the third pattern (AILTTLANWARKFL). In contrast to “16890198” its content is related implicitly to pattern 3. Their precision scores are 93% and 28%, respectively.

*Pattern 4*. The ISAP approach found that 10% of the articles in the whole collection have the fourth pattern “AKKVFKRLEKLFSKIQNDKL.”

*Pattern 5*. The fifth pattern “ALSILRGLEKLAKMGIALTNCKATKKC” is found in only a small sample of the articles that have antibacterial peptides.

**Figure 3:**
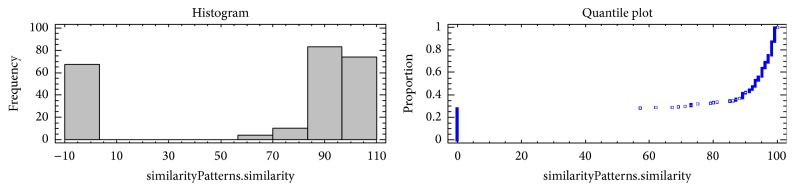
A distribution of the antibacterial-peptides patterns according to their precision.

A distribution of the antibacterial-peptides patterns according to their precision is shown in Figure 3.

Nine classes of the antibacterial-peptides patterns were grouped according to their precision as shown in Table 8. The average of accuracy is 67.0756, the standard deviation is 42.5447, and the coefficient of variation is 63.4278% in a range of 100.0 for 238 antibacterial-peptides patterns.

**Figure 4:**
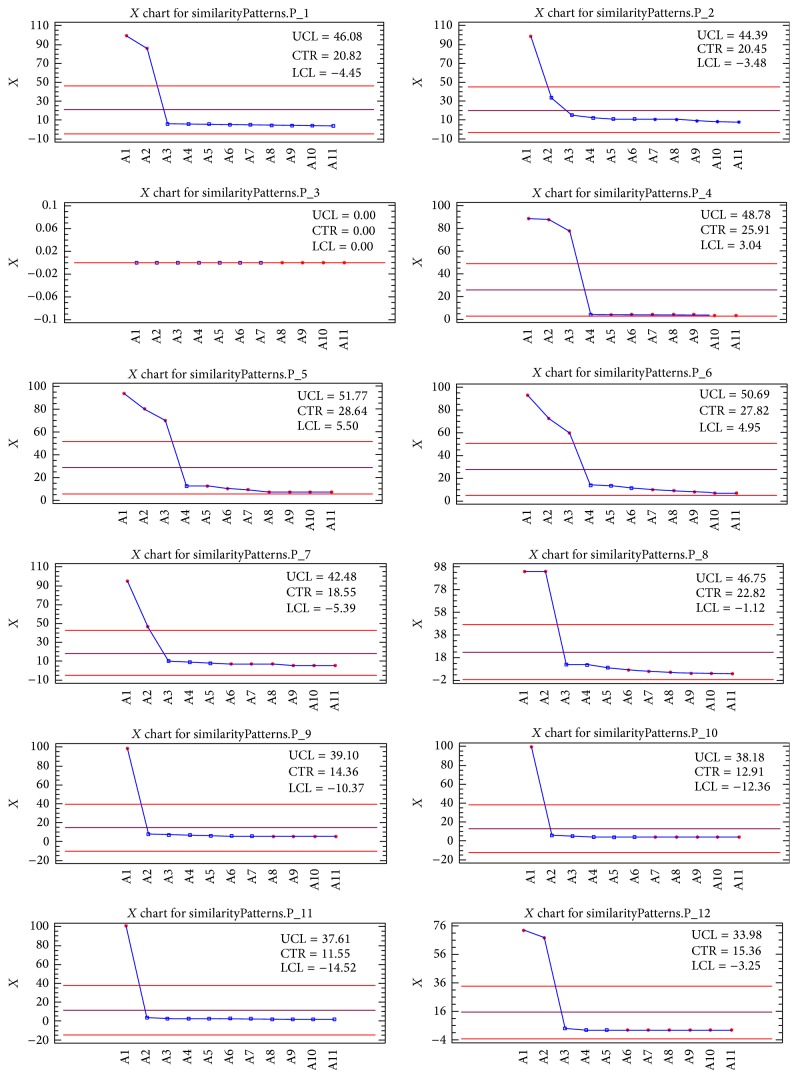
Twelve antibacterial-peptides patterns and their first eleven research articles related to their precision.

The first twelve antibacterial-peptides patterns and their first eleven research articles related to their precision are shown in Figure 4. For example, pattern 2 has the highest precision with 98% where the article corresponds to PMID 23069634.

## 5. Discussion

All units of knowledge that researchers have been published in different periods are heterogeneous and with very different styles.

The ISAP approach extracted latent relationships from peptide articles with antibacterial activity that can be used to identify new relationships on an experimental basis.

Furthermore, the ISAP approach allows us to find relevant subsets of papers based on antibacterial peptides but that have not been annotated manually.

Some of the results of the ISAP approach are as follows: (1) an annotated list with antibacterial peptides activity and their PMIDs was provided; (2) a lexicon of terms based on antibacterial peptides was established.

Abstracts that are not related to our experiments were the antibacterial peptides annotated manually and not published in Tossi Database. This represents 20% of the collection. In this respect, there is an opportunity window for anyone who wants to experiment in their laboratory and report their results to the community.

Finally, it is recommended to use controlled vocabularies for the process of writing the articles so as to facilitate the classification and search process. Future research will use GPUs in order to accelerate the compute process.

## 6. Conclusion

ISAP was trained by supervised learning and LSA technique in order to learn from the abstracts that have antibacterial peptides.

In the phase of discovery, it is possible to locate all items that match the search although they are not sequences in the abstract. This is valuable because the researchers published their findings in very heterogeneous ways and databases related to peptides that are not up to date. In addition, the articles production is exponential and performing the search manually is not possible.

Through a blind approach it is possible identify if a peptide is antibacterial as well as to discover new peptides that serve to develop the next generation antibiotics.

Moreover, personalized medicine promises new treatment and the eradication of diseases until today has not been solved such as the case of cancer.

ISAP founds that 35% of the abstracts sample had antibacterial peptides and we tested in the updated Antimicrobial Peptide Database 2 (APD2) and its application in peptide design reaching a correlation value of 1.

ISAP predicted that 45% of the abstracts had antibacterial peptides. That is, ISAP found that 810 antibacterial peptides were not classified like that, so they are not reported in APD2. As a result, this new search tool would complement the APD2 database with a set of peptides that are candidates to be antibacterial but which have not been tagged as such.

ISAP discovered that 20% of the abstracts were not semantic related to ISAP, so it concludes that it did not contain antibacterial peptides and we verified that into APD2. Our experiments proved to have a correlation of one between the results of the ISAP model and the APD2.

## Figures and Tables

**Box 1:**
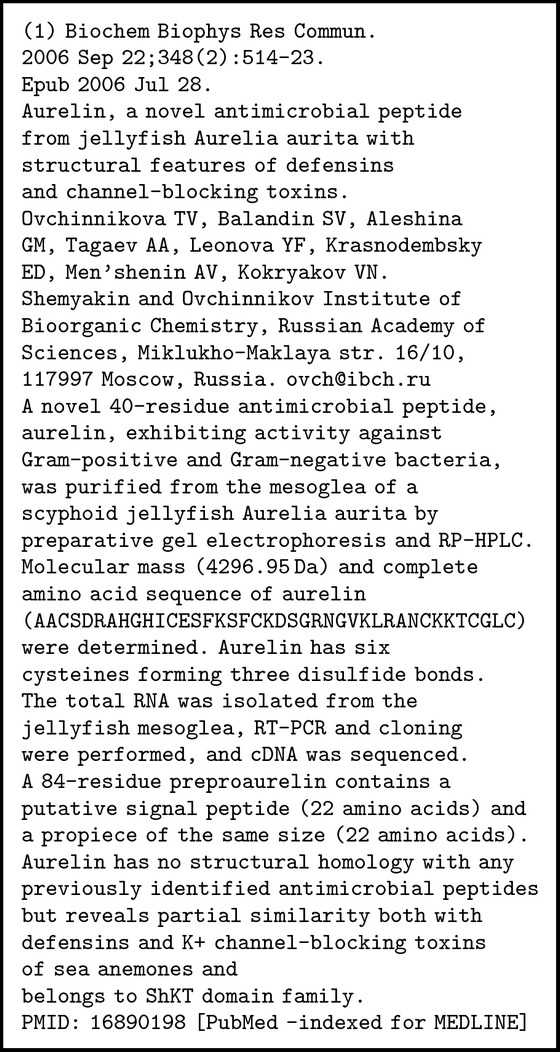
One example of a pattern abstract.

**Table 1:** Some of the 1762 antibacterial-peptides patterns.

ID	Peptide
AP00001	GLWSKIKEVGKEAAKAAAKAAGKAALGAVSEAV
AP00002	YVPLPNVPQPGRRPFPTFPGQGPFNPKIKWPQGY
AP00004	NLCERASLTWTGNCGNTGHCDTQCRNWESAKHGACHKRGNWKCFCYFDC
AP00005	VFIDILDKVENAIHNAAQVGIGFAKPFEKLINPK
AP00006	GNNRPVYIPQPRPPHPRI
AP00007	GNNRPVYIPQPRPPHPRL
AP00008	RLCRIVVIRVCR
AP00009	RFRPPIRRPPIRPPFYPPFRPPIRPPIFPPIRPPFRPPLGPFP
AP00010	RRIRPRPPRLPRPRPRPLPFPRPGPRPIPRPLPFPRPGPRPIPRPLPFPRPGPRPIPRPL
AP00011	WNPFKELERAGQRVRDAVISAAPAVATVGQAAAIARG
AP00012	GLFDIIKKIAESI
AP00013	GLFDIIKKIAESF
⋮	⋮
AP02172	FFGSLLSLGSKLLPSVFKLFQRKKE

**Table 2:** A partial view of the terms-abstracts matrix was generated with 8892 terms that contain data related to the antibacterial-pattern abstract collection.

Terms	*a*_1_	*a*_2_	*a*_3_	*a*_*n*_
antimicrobi	0.0735581075541086	− 0.0244847301347335	− 0.00710470395642022	− 0.0252782143833409
peptid	0.0215719016953859	− 0.00659599218340233	0.00526833571530154	− 0.0027931356297511
identifi	0.0778285095429026	0.0363743255478556	− 0.0125611882021721	0.0335513355175925
puparum	0.0031890062317054	− 0.00433904701191521	0.000278800242086269	− 0.000536875590521908
cdna	0.0752034473971184	− 0.100948929444321	− 0.120231206062553	0.00808336458293407
clone	0.0506850130947056	− 0.0638498278798138	− 0.0757289501422613	0.00855381871117201
shen	0.0015945031158527	− 0.0021695235059576	0.000139400121043145	− 0.000268437795260781
cheng	0.00269501661729059	− 0.00524105545332141	0.0058427721000811	0.00350622976781349
altosaar	0.0015945031158527	− 0.0021695235059576	0.000139400121043145	− 0.000268437795260801
state	0.0113803981224781	− 0.0145520357690956	0.0142282472637731	− 0.000138326518028741
kei	0.0197729657477364	− 0.0367429529088927	− 0.0172473645432955	0.000106625559641722
laboratori	0.0262664068350282	− 0.0268798478912976	− 0.0148428311950068	− 0.0498138841648604
rice	0.0015945031158527	− 0.0021695235059576	0.000139400121043145	− 0.000268437795260802
biologi	0.0310863666985836	− 0.027616438954597	0.00141517510229895	− 0.0374922667242602
ministri	0.00401909815031002	− 0.00553150270576137	− 0.00244184305151988	− 0.00110105794405476
agricultur	0.0196836003644828	− 0.0365550561462568	− 0.0376858882953384	0.00278678534624657
molecular	0.0410216058415589	− 0.037285345518391	0.00109576583310193	− 0.0519376532603683
crop	0.0015945031158527	− 0.0021695235059576	0.000139400121043145	− 0.000268437795260802
pathogen	0.0276770473353559	0.00130713009997107	0.0051845473156494	− 0.00667894125821291
insect	0.00541558702390805	− 0.00725187831084491	0.00234699169994147	− 0.00139023706808898
*t*_*n*_	X	X	X	X

**Table 3:** Total average accuracy of classification is 97% and misclassification or error rate is 2.11%.

Correctly classified instances	1762 (97.8889%)
Incorrectly classified instances	38 (2.1111%)
Kappa statistic	0.9666
Mean absolute error	0.0269
Root mean squared error	0.1161
Relative absolute error	6.3555
Root relative squared error	25.2452
Total number of instances	1800

**Table 4:** Detailed accuracy by class.

TP rate	FP rate	Precision	Recall	*F*-measure	ROC area	Class
0.94	0	1	0.94	0.969	0.973	antibacterial
1	0	1	1	1	1	noAntibacterial
1	0.038	0.955	1	0.977	0.975	canBeAntibacterial

**Table 5:** The confusion matrix.

*a*	*b*	*c*	Classified as
592	0	38	*a* = antibacterial
0	360	0	*b* = noAntibacterial
0	0	810	*c* = canBeAntibacterial

**Table 6:** Five peptides patterns selected randomly.

ID	Peptide
Pattern 1	AACSDRAHGHICESFKSFCKDSGRNGVKLRANCKKTCGLC
Pattern 2	GLFDVIKKVASVIGGL
Pattern 3	AILTTLANWARKFL
Pattern 4	AKKVFKRLEKLFSKIQNDK
Pattern 5	ALSILRGLEKLAKMGIALTNCKATKKC

**Table 7:** As a result for the second pattern the following research articles obtained in a list recorded in descending order.

PMID	Title	Percentage
23069634	Structural and activity changes in three bioactive anuran peptides when Asp is replaced by isoAsp.	98.116781%

11478963	Enhancing the hypotensive effect and diminishing the cytolytic activity of hornet mastoparan B by D-amino acid substitution.	13.075785%

15917539	Proline conformation-dependent antimicrobial activity of a proline-rich histone h1 N-terminal peptide fragment isolated from the skin mucus of Atlantic salmon.	10.655851%

16470724	Host-defence skin peptides of the Australian streambank froglet *Crinia riparia*: isolation and sequence determination by positive and negative ion electrospray mass spectrometry.	9.804270%

9231329	Hydrophobic effects on antibacterial and channel-forming properties of cecropin A-melittin hybrids.	8.624214%

⋮	⋮	

**Table 8:** Nine classes of the antibacterial peptides patterns according to their precision. Frequency tabulation for similaritypatterns.similarity.

Class	Lower limit	Upper limit	Midpoint	Frequency	Relative frequency	Cumulative frequency	Cum. rel. frequency
	at or below	−10.0		0	0.0000	0	0.0000
1	−10.0	3.33333	−3.33333	67	0.2815	67	0.2815
2	3.33333	16.6667	10.0	0	0.0000	67	0.2815
3	16.6667	30.0	23.3333	0	0.0000	67	0.2815
4	30.0	43.3333	36.6667	0	0.0000	67	0.2815
5	43.3333	56.6667	50.0	0	0.0000	67	0.2815
6	56.6667	70.0	63.3333	4	0.0168	71	0.2983
7	70.0	83.3333	76.6667	10	0.0420	81	0.3403
8	83.3333	96.6667	90.0	83	0.3487	164	0.6891
9	96.6667	110.0	103.333	74	0.3109	238	1.0000
	above	110.0		0	0.0000	238	1.0000

Mean = 67.0756; standard deviation = 42.5446.
